# Sex and Gender Medical Education Summit: a roadmap for curricular innovation

**DOI:** 10.1186/s13293-016-0091-9

**Published:** 2016-10-14

**Authors:** Eliza L. Chin, Marley Hoggatt, Alyson J. McGregor, Mary K. Rojek, Kimberly Templeton, Robert Casanova, Wendy S. Klein, Virginia M. Miller, Marjorie Jenkins

**Affiliations:** 1 American Medical Women‘s Association, University of California San Francisco, San Francisco, CA USA; 2 Laura W. Bush Institute for Women‘s Health, Texas Tech University Health Sciences Center, Amarillo, TX USA; 3 Division of Sex and Gender in Emergency Medicine, Warren Alpert Medical School of Brown University, Providence, RI USA; 4 Center for Urban Research and Learning, Loyola University Chicago, Chicago, IL USA; 5 American Medical Women‘s Association, Orthopedic Surgery, University of Kansas School of Medicine, Kansas City, KS USA; 6 Obstetrics and Gynecology, Texas Tech University Health Sciences Center, Lubbock, TX USA; 7 VCU Institute for Women‘s Health, Virginia Commonwealth University School of Medicine, Richmond, VA USA; 8 Women‘s Health Research Center, Mayo Clinic, Rochester, MN USA

## Abstract

The Sex and Gender Medical Education Summit: a roadmap for curricular innovation was a collaborative initiative of the American Medical Women's Association, Laura W. Bush Institute for Women’s Health, Mayo Clinic, and Society for Women's Health Research (www.sgbmeducationsummit.com). It was held on October 18–19, 2015 to provide a unique venue for collaboration among nationally and internationally renowned experts in developing a roadmap for the incorporation of sex and gender based concepts into medical education curricula. The Summit engaged 148 in-person attendees for the 1 1/2-day program. Pre- and post-Summit surveys assessed the impact of the Summit, and workshop discussions provided a framework for informal consensus building. Sixty-one percent of attendees indicated that the Summit had increased their awareness of the importance of sex and gender specific medicine. Other comments indicate that the Summit had a significant impact for motivating a call to action among attendees and provided resources to initiate change in curricula within their home institutions. These educational efforts will help to ensure a sex and gender basis for delivery of health care in the future.

## Background

Sex and gender based medicine (SGBM) is the science of similarities and differences in the human biology of men and women, both in health and disease. This field has its roots in the women’s health movement but has gone further to consider the biology and pathophysiology of disease as well as the sociocultural influences for both women and men. A primary impetus for the emergence of SGBM was the increasing awareness that research conducted with white males might not apply to women or other ethnic groups [1, 2]. As a result, the 1993 National Institutes of Health (NIH) Revitalization Act mandated that researchers include both women and minorities in clinical research [3]. Though studies now include women, differences in outcomes are not consistently assessed or reported by sex, making it difficult to know how, or if, related recommendations can or should be applied to either sex.

A 2001 Institute of Medicine (IOM) report emphasized that sex-based differences were due to more than hormonal differences and that “every cell has a sex” [2]. Subsequently, both the Federal Drug Administration (FDA) and NIH have expanded requirements that both vertebrate and human research include males and females and that collective data should be analyzed by sex as an independent variable. In addition, the sex of isolated or cultured cells should be identified. The report also clarified the terminology “sex” and “gender.” In broad terms, sex is a biological construct where living things are characterized as male or female according to chromosomal complement and reproductive organs [4]. Gender refers to a person’s self-representation and behaviors as man or woman within the context of social structure and culture [5, 6]. Sex and gender are interrelated in terms of health and illness, such that one’s social environment and behaviors, both of which are gendered, influence one’s biology. For example, both men and women with acute coronary syndrome (ACS) often present with chest pain but their descriptions of pain and associated symptoms may vary, demonstrating sex differences in the pathophysiology of ACS and gender variations in reporting [7, 8]. Both variables must be considered in research as well as in medical education and practice.

Despite progress in women’s health research, the IOM report indicated that significant gaps remained in the application of research findings to improve patient care [2]. Applying the findings from research conducted in men to the clinical care of women has contributed to gender disparities in healthcare [9, 10]. These disparities result from biological differences in etiology and presentation of disease, differences in pharmacokinetics leading to ineffective treatment or drug toxicity, or conscious or unconscious gender bias in the physician-patient interaction [11–13].

These gaps demonstrate the need for additional research but also the need for the inclusion of sex and gender based medical concepts in all levels of health professional curricula. The majority of US medical schools do not have a formal sex and gender specific integrated medical curriculum [14]. Therefore, educational reform will be a key factor in shifting this paradigm. Topics included under the rubric of “women’s health” or “men’s health” can no longer be limited to reproductive issues or only those conditions that can be observed in a single sex, e.g., prostate cancer. Rather, SGBM in medical education must include a discussion of similarities and differences between sexes and genders in the etiology, risk factors, prevention, presentation, and response to treatment for all health conditions. It is within this context that the Sex and Gender Medical Education Summit was planned.

## Methods

### Conference planning

In 2012, a 2 day workshop was convened at the Mayo Clinic with leaders from 13 medical and public health institutions, governmental agencies, and the Canadian Institute of Health and Gender (Table 1) to discuss the need for integrating SGBM into medical education and training, as well as to develop implementation strategies to bring about this change. Recommendations from the workshop addressed institutional engagement and the need to provide teaching materials that could readily be integrated into established curricula [15].

**Table 1:** Attendees of the 2012 Mayo Clinic 2 day workshop on “Embedding Concepts of Sex and Gender Health Differences into Medical Curricula”

Name	Affiliation
Carl F. Anderson, MD	Mayo Clinic
Delia M. Camacho, PhD	School of Health Professions University of Puerto Rico Medical Sciences
Janine Austin Clayton, MD	Director, Office of Research for Women’s Health, National Institute of Health
Shivani Dhawan, BS	Cedars-Sinai Heart Institute, Women’s Heart Center
Richard Dickerson, PhD	Texas Tech University Health Sciences Center
Priscilla M. Flynn, DrPH	School of Dentistry, University of Minnesota
Salma Iftikhar, MD	Mayo Clinic
Marjorie Jenkins, MD, MEHP	Professor of Medicine, Division of Women’s Health and Gender-Specific Medicine, Texas Tech University Health Sciences Center
Jani R. Jensen, MD	Mayo Clinic
Joy Johnson, PhD, RN, FCAHS	Scientific Director, Institute of Gender and Health, Canadian Institutes of Health Research University of British Columbia
Sabrina A. Matoff-Stepp, PhD	Director, HRSA Office of Women’s Health
Bradley B. Miller, MD	Texas Tech University Health Sciences Center
Virginia M. Miller, PhD	Professor, Surgery and Physiology, Mayo Clinic Immediate Past President of the Organization for the Study of Sex Differences
Ana E. Núñez, MD	Director of the Center of Excellence and Women’s Health Education Program, Drexel University College of Medicine
Cheri L. Olson, MD	Mayo Clinic Health System
Limor Raz, PhD	Mayo Clinic
CDR Morrisa Rice, MHA	Senior Public Health Analyst HRSA Office of Women’s Health
Jane F. Reckelhoff, PhD	University of Mississippi Medical Center,Women’s Health Research Center
April E. Ronca, PhD	Wake Forest School of Medicine
Matthew A. Saracusa	Cedars-Sinai Medical Center
Londa Schiebinger, PhD	John L. Hinds Professor of History of Science Director of the EU/US Gendered Innovations in Science, Health and Medicine, and Engineering Project, Stanford University
Lynne T. Shuster, MD	Director, Office of Women’s Health Consultant, Women’s Health Clinic Mayo Clinic
Thomas R. Viggiano, MD, MEd	Associate Dean for Faculty Affairs at Mayo Medical School, Professor, College of Medicine, Mayo Clinic
Janet Vittone, MD	Consultant in General Internal Medicine Mayo Clinic
Janice Werbinski, MD, FACOG	Michigan State U College of Human Medicine
Susan F. Wood, PhD	Associate Professor of Health Policy Director of the Jacobs Institute of Women’s Health George Washington University School of Public Health and Health Services
Kimberly Templeton, MD	University of Kansas

Adapted from Miller VM, Rice M, Schiebinger L, Jenkins MR, Werbinski J, Nunez A, Wood S, Viggiano TR, Shuster LT, Embedding Concepts of Sex and Gender Health Differences into Medical Curricula, J Womens Health 22(3), page 201, 2013 [15, Appendix 1]

In 2014, the American Medical Women’s Association (AMWA) and the Laura W. Bush Institute for Women’s Health (LWBIWH) convened a planning group (Table 2) to develop a Sex and Gender Medical Education (SGME) Summit for the purpose of increasing SGBM education on a national scale and ensuring that the next generation of physicians would be competent in this field. Leaders from medical school institutions and professional associations were invited to join a senior advisory committee (Table 3) to provide input on the Summit program. Initial objectives for the Summit were to (a) review the current climate of sex and gender education in medical schools, (b) provide curricular resources for schools of medicine, (c) align SGBM with required Liaison Committee on Medical Education (LCME) Accreditation Standards, and (d) identify present and future needs in closing these gaps in medical education. Mayo Clinic was chosen as the host site and CME provider, with the AMWA, the LWBIWH, and the Society for Women’s Health Research (SWHR) as joint providers.

**Table 2:** SGME Summit planning committee members

Planning committee members	
Marjorie Jenkins, MD, MEHP, FACP (Chair)	Professor of Medicine and Chief Scientific Officer Laura W. Bush Institute for Women’s Health Texas Tech University Health Sciences Center
Eliza Lo Chin, MD, MPH, FACP (Co-Chair)	Executive Director American Medical Women’s Association Assistant Clinical Professor of Medicine University of California, San Francisco
Virginia Miller, PhD (Host Co-chair)	Professor and Director, Women’s Health Research Center, Mayo Clinic
Robert Casanova, MD	Assistant Dean of Clinical Sciences Curriculum Texas Tech University Health Sciences Center
Wendy S. Klein, MD, MACP	Associate Professor Emeritus, Virginia Commonwealth University School of Medicine
Alyson J. McGregor, MD, MA, FACEP	Director, Division of Sex and Gender in Emergency Medicine Associate Professor of Emergency Medicine Warren Alpert Medical School of Brown University
Kimberly Templeton, MD	Professor of Orthopedic Surgery and Health Policy and Management, University of Kansas School of Medicine, President-elect, American Medical Women's Association
Jan Werbinski, MD, FACOG	Executive Director Sex and Gender Women’s Health Collaborative

**Table 3:** SGME Summit senior advisory committee members

Senior advisory committee members	
Steven L. Berk, MD	Provost and Dean Texas Tech University Health Sciences Center
Humayun J. Chaudhry, DO, MS, MACP, FACOI	President and CEO Federation of State Medical Boards
Phyllis E. Greenberger, MSW	President and CEO, Society for Women’s Health Research
John C. Jennings, MD	Professor of Medicine, Texas Tech University Health Sciences Center, Permian Basin
Cynda Ann Johnson, MD, MBA	President and Dean, Virginia Tech Carilion School of Medicine and Research Institute
Jose Manuel De La Rosa, MD	Provost and Vice President for Academic Affairs Paul L. Foster School of Medicine Texas Tech University Health Sciences Center
Tedd Mitchell, MD	President Texas Tech University Health Sciences Center
Theresa Rohr-Kirchgraber, MD, FACP	President, American Medical Women’s Association
Robert D. Simari, MD	Executive Dean University of Kansas School of Medicine
Connie Tyne, MS	Executive Director Laura W. Bush Institute for Women's Health
Steven E. Weinberger, MD, FACP	Executive Vice President and Chief Executive Officer American College of Physicians

Medical schools and osteopathic schools in the USA and Canada were invited to send a representative to the Summit. Engagement occurred through a combination of email invitations, letters, phone calls, announcements through the Association of American Medical Colleges (AAMC), and grassroots efforts. To encourage participation, educational grants were provided to cover registration and lodging for one designated representative from each participating institution. An effort was made to recruit key faculty who would be instrumental in developing, implementing, and assessing outcomes of medical curricula at their institutions.

### The SGME Summit

The 1 1/2 day program included a keynote address, ten educational sessions, two panel discussions, a poster session, and two concurrent workshops (Table 4). The Summit faculty included nationally renowned SGBM experts as well as leaders in medical education and curriculum development (Table 5). The panel discussions, with representatives from the U.S. and international institutions, highlighted the different methodologies and models for integrating SGBM content into medical education, for example, a fully integrated curriculum or adoption of a module that students could complete online. The poster sessions allowed individuals to display and discuss their work with other attendees. The workshops considered two topics—utilization of SGBM resources in medical schools and SGBM student competencies. Attendees selected which workshop they wanted to attend. In conjunction with a facilitator, they discussed the topic and developed consensus points for each group which were reported back to the larger group. Pre- and post-tests were disseminated electronically to document attendees’ experience and knowledge in SGBM.

**Table 4:** SGME Summit agenda

Summit agenda	
Sunday, October 18, 2015	
Keynote: Taking Sex and Gender from the Bench to the Bedside Requires the Classroom	
Sex and Gender Medicine - What It Is and What It Isn’t	
International Sex and Gender Curriculum Panel and Discussion	
Poster Session	
Monday, October 19, 2015	
Sex and Gender in Research and Education: The Federal Landscape	
Sex and Gender in Medicine: Patient and Provider Considerations	
What Students Think about Sex and Gender Based Medicine: Results of a National Climate Survey	
Where to Go When You Want to Know – Sex and Gender Based Medicine Education Resources	
Lessons from the Field: Models of Sex and Gender Based Curricula	
Avoiding the Shoehorn: Strategies for Incorporating New Curricular Content	
Integrating New Curricular Content: Think Assessment First	
Introduction of an LGBT Curriculum at the University of California, San Francisco	
Sex and Gender Based Medicine in Interprofessional Education: Putting it All Together	
Workshop A: Utilization of SGBM Resources in U.S. Medical Schools: Overcoming Barriers to Achieve Action	
Workshop B: Creating SGBM Student Competencies in Alignment with the AAMC	
From Roadmap to Reality: Your Role as a Change Agent

**Table 5:** SGME Summit speakers

Speakers and contributors
Bethany Applebaum, MPH, MA Public Health Analyst Health Resources and Services Administration (HRSA) US Department of Health and Human Services
C. Noel Bairey Merz, MD, FACC, FAHA Women’s Guild Endowed Chair in Women’s Health Director, Barbra Streisand Women’s Heart Center Director, Linda Joy Poling Women’s Heart Health Program Director, Preventive Cardiac Center Professor of Medicine, Cedars-Sinai Heart Institute
Jabbar R. Bennett, PhD Associate Provost, Diversity and Inclusion Associate Professor of Medicine Feinberg School of Medicine, Northwestern University
Richard A. Berger, MD, PhD Professor of Orthopedic Surgery and Anatomy Dean, Mayo School of Continuous Professional Development Medical Director, Mayo Clinic Online Learning
Ann Bonham, PhD Chief Scientific Officer Association of American Medical Colleges
Ruth Bush, MD, JD, MPH Vice Dean for Academic Affairs Professor of Surgery Texas A&M Health Science Center College of Medicine
Robert Casanova, MD Assistant Dean of Clinical Sciences Curriculum Associate Professor, Program Director Obstetrics and Gynecology Texas Tech University Health Sciences Center, Lubbock
Eliza Lo Chin, MD, MPH, FACP Summit Co-Chair Executive Director, American Medical Women’s Association Assistant Clinical Professor of Medicine University of California, San Francisco
Terri L. Cornelison, MD, PhD, FACOG Associate Director for Clinical Research Captain, United States Public Health Service Office of Research on Women’s Health (ORWH) National Institutes of Health
Gillian Einstein, MD Associate Professor, Department of Psychology Dalla Lana School of Public Health, University of Toronto Director, Collaborative Graduate Program in Women’s Health Visiting Professor of Neuroscience & Gender Medicine Linköping University, Sweden
Phyllis Greenberger, MSW President and CEO Society for Women’s Health Research
Marjorie Jenkins, MD, MEHP, FACP Summit Chair, Professor of Medicine Chief Scientific Officer, Laura W. Bush Institute for Women’s Health Co-Director, Sex and Gender Curriculum Program Texas Tech University Health Sciences Center
Jani R. Jensen, MD Assistant Professor, Obstetrics and Gynecology Chair, Curriculum Development Mayo Medical School
Georgios Kararigas, PhD DZHK W1 Professor Institute of Gender in Medicine Charité University Hospital
Karolina Kublickiene, MD, PhD Associate Professor of Obstetrics and Gynecology CEO, Center of Gender Medicine Senior Scientist Department of Clinical Science Intervention and Technology Karolinska Institutet
Marianne J. Legato, MD, FACP Emerita Professor of Clinical Medicine Columbia University
John Luk, MD Assistant Dean for Interprofessional Integration Assistant Professor of Medicine Dell Medical School at University of Texas at Austin
Alyson J. Mcgregor, MD, MA, FACEP Director, Division of Sex and Gender in Emergency Medicine Associate Professor of Emergency Medicine Warren Alpert Medical School of Brown University
Alex Jeffrey Mechaber, MD Professor of Medicine Senior Associate Dean for Undergraduate Medical Education University of Miami Miller School of Medicine
Bonnie M. Miller, MD Senior Associate Dean for Health Sciences Education Associate Vice Chancellor for Health Affairs Professor of Medical Education Administration Vanderbilt University School of Medicine
Virginia Miller, PhD Professor, Departments of Physiology and Surgery Director, Women’s Health Research Center Mayo Clinic, Rochester MN
Ana E. Núñez, MD Professor of Medicine Associate Dean of Urban Health Equity, Education and Research Director, Women’s Health Education Program and National Center of Excellence in Women’s Health, Drexel University College of Medicine
Janet Pregler, MD Professor of Clinical Medicine Director, Iris Cantor - UCLA Women’s Health Center David Geffen School of Medicine at University of California, Los Angeles
Patricia A. Robertson, MD Inaugural Member, Haile T. Debas Academy of Medical Educators Professor, Department of Obstetrics, Gynecology and Reproductive Sciences, University of California San Francisco School of Medicine
Pamela E. Scott, PhD, MA Director, Research and Development Office of Women’s Health Food and Drug Administration (FDA) / Office of the Commissioner
Connie Tyne, MS Executive Director Laura W. Bush Institute for Women’s Health
Jan Werbinski, MD, FACOG Associate Clinical Professor of Obstetrics and Gynecology Western Michigan University Homer Stryker School of Medicine Executive Director, Sex and Gender Women’s Health Collaborative

### Post-Summit work

Following the Summit, a toolkit and detailed summary proceedings were disseminated electronically and in print to all attendees, participating institutions, supporting organizations, national medical associations, and individuals in other networks. A work group was convened to develop a set of sex and gender medical student competencies. Follow-up surveys were developed to assess the impact of the Summit on the advancement of SGBM within medical education curricula.

## Results

### Attendees

Attendees (*n* = 148: 119 females, 29 males) represented the spectrum of health and research credentials (Table 6).

**Table 6:** SGME Summit attendees

Designation	Number of participants
PhD	37
MD	90
DO	5
MPH	10
Medical student	10

(*n* = 148, female = 119, male = 29). Note: Some participants had dual degrees

### Participants’ knowledge and attitudes

Results of the Summit were based on pre- and post-Summit surveys. A pre-test was made available to participants via email before the Summit. A post-test was distributed via email after the Summit. Sixty-seven participants completed the pre-test, and 62 (unmatched) participants completed the post-test. These assessments were comprised of yes/no and Likert scale questions. They were intended to ascertain participants’ attitudes and knowledge of SGBM and level of SGBM education currently in place at participants’ institutions. The final questions assessed participants’ satisfaction with the Summit itself, including interest in attending a second event. The participants were also able to provide open-ended comments about their Summit experience.

Participants’ familiarity with the topic of sex and gender differences in health and diseases increased from 81 % in the pre-test to 93 % in the post-test (strongly agree/agree). When asked if they believed the FDA should consider recommending dosages based on the sex of the patient, 69 % of the participants agreed (strongly agree/agree) on the pre-test and 97 % agreed (strongly agree/agree) on the post-test, an increase of 28 %. One of the most dramatic attitudinal shifts was in participants’ response to the statement “Sex and gender based medicine is a fundamental aspect of precision medicine.” Forty percent of the respondents strongly agreed in the pre-test, while 81 % strongly agreed on the post-test, an increase of 41 % (Table 7) [16].

**Table 7:** SGME participant survey responses

	Pre (%)	Post (%)
I am familiar with the topic of sex and gender differences in health and disease.		
Strongly disagree	0	0
Disagree	4.5	5.1
Neutral	14.9	1.7
Agree	59.7	42.4
Strongly agree	20.9	50.8
The FDA should consider recommending dosages based on the sex of the patient.		
Strongly disagree	0	0
Disagree	3	0
Neutral	28.3	3.4
Agree	41.8	30.5
Strongly agree	26.9	66.1
Sex and gender-based medicine is a fundamental aspect of precision medicine.		
Strongly disagree	0	0
Disagree	0	0
Neutral	9	3.5
Agree	50.7	15.8
Strongly agree	40.3	80.7
Has this conference changed your opinion of the importance of sex and gender-specific health?		
Yes	–	61
Somewhat	–	22
No	–	17

Note: Pre- and post-test responses were unmatched. This data was also presented in the Summit Proceedings [16]

### Workshop outcomes

Concurrent workshops were conducted in an effort to establish the framework necessary for the successful creation of national medical student competencies in SGBM. Workshop A, “Utilization of SGBM Resources in U.S. Medical Schools: Overcoming Barriers to Achieve Action,” focused on participants’ input regarding experiences at their corresponding institutions with novel curricular integration and implementation. The participants were given pre-work assignments which included questions regarding each individual’s experiences with initiating educational projects at their own institution and recommended strategies for incorporating SGBM. Although no formal consensus building process was utilized, the workshops provided a framework for discussion. The ensuing discussion resulted in three common themes: (1) participants felt strongly that SGBM material should be presented as a longitudinal curriculum thread woven into existing educational materials, (2) faculty development was necessary along with a multifaceted approach for integrating SGBM into existing educational materials, and (3) developing an advisory committee comprised of medical school curriculum experts to oversee the process was integral to success.

Workshop B, “Creating SGBM Student Competencies in Alignment with the Association of American Medical Colleges (AAMC),” included discussions of how best to approach development of a set of competencies in SGBM. Pre-work assignments were comprised of questions to facilitate approaches to generating SGBM competencies and strategies for their formulation. The discussion revealed broad consensus that SGBM curricula should encompass all health conditions, include both basic and clinical sciences, and utilize existing curricular components in women’s health, emergency medicine, and lesbian, gay, bisexual, transgender (LGBT) competencies because these have already been defined and overlap with concepts of sex and gender in a breadth of body systems. Engaging stakeholders such as students and faculty would be essential to attaining sustainable integration.

Conclusions from each workshop were then presented to the larger group. SGBM curricular integration, application, and synthesis must generate measurable objectives; therefore, ongoing evaluation strategies are necessary. The participants suggested using a theoretical framework to assess competency such as Miller’s pyramid (knows, knows how, shows how, and does) to cover multiple competency levels and monitor the progressive achievement of measurable milestones. Workshop logistics, clear definitions and terminology, approaches to competency development, and a table outlining overall implementation strategies are presented and further discussed in an accompanying manuscript “Utilization of Sex and Gender Based Medical Education Resources and Creating Student Competencies: A Summit Workshop Summary” [17].

### Participant response to the Summit

The Summit participants were asked “Has this conference changed your opinion of the importance of sex and gender-specific health?” On the post-test, 61 % of the participants responded “Yes,” 22 % responded “Somewhat,” and 17 % responded “No.” This indicates that the Summit had an impact on the views of the vast majority of attendees.

Table 8 includes participants’ comments that demonstrate the impact and the role of the Summit in serving as a call to action. Several participants outlined concrete plans for advancing SGBM in their medical school’s curriculum.

**Table 8:** Comments from SGME participants

Comments from participants
“I will develop a proposal for our curriculum committee that we include sex and gender-specific material in all our courses and clerkships…I will also request that student assessments include items about sex- and gender-based differences.”
“I plan to meet with individual course coordinators to review what sex-and gender-specific health topics are currently included in each course and discuss how additional sex- and gender-specific health topics can be integrated within each course. The resources that were made available to summit participants are outstanding, and they will facilitate the promotion of additional curricular emphasis of this area.”
“We will be presenting information learned from the meeting to the next Dean’s Circle and including some of the fast facts in all of our women’s health lectures.”
“I will be meeting with the Associate Deans of Clinical Sciences and Basic Sciences to discuss suggestions of integrating sex and gender slides and information through specific content lectures.”

#### Recurring themes

Throughout the Summit, there appeared to be several recurring themes. The three that stand out as central to success were (1) overcoming preconceived notions about sex and gender, (2) the need for time and resources, and (3) increasing awareness.

In order to successfully implement meaningful curricular change, the administration, faculty, and learners must overcome longstanding conscious and unconscious bias about SGBM issues. Sex as a biological variable cannot be overlooked as it influences all aspects of health. While the spectrum of Lesbian, Gay, Bisexual, Transgender, Queer, Intersex, Asexual (LGBTQIA) health is an integral part of the dialogue, SGBM represents a much broader umbrella that encompasses a gender-based approach to all aspects of individualized care.

Medical education institutions and faculty face limitations of curricular time and resources. They would find it helpful to utilize existing content such as the Texas Tech University Health Sciences Center PubMed Search Tool and Slide Library, as well as other tools available at sites such as the Sex and Gender Women’s Health Collaborative (http://www.sgwhc.org). Time issues are compounded by the limited curricular space available for incorporating new content and the complexities of “curricular reform.” Threading SGBM concepts throughout current curricula might be a more effective and pragmatic approach, as demonstrated by the successful program at Charité Hospital in Germany [18].

Increasing SGBM awareness involves engaging all stakeholders: health professions’ school leadership, researchers, instructors, learners, and the public. This approach has been implemented at the Alpert Medical School of Brown University’s Sex and Gender in Emergency Medicine Division. This program has focused on “advanced care through person specific education and advocacy” and has used public service posters to prepare patients for a personalized emergency department experience.

Ultimately, all of these issues require a faculty champion or “change agent” who can drive curricular integration and serve as a resource. It is imperative to support these individuals’ training by sponsoring attendance at national conferences where they can gain content knowledge and establish a network of like-minded individuals.

John Kotter’s “8-Step Process for Leading Change” [19] can be adapted and serve as a useful guide:

1. Establish a sense of urgency by stressing the patient care aspect of SGBM and its immediate impact on personalized medicine
2. Create a guiding coalition including researchers, instructors, learners and patients
3. Develop a clear shared vision by accessing and building upon existing resources
4. Communicate the vision through events such as the SGME Summit
5. Empower people to act upon the vision by recruiting other like-minded individuals
6. Create short term wins
7. Consolidate and build on the gains by facilitating dissemination
8. Institutionalize the change by developing core competencies in SGBM anchored to AAMC competencies

## Discussion

The impact and scope of SGBM on patient care needs to be recognized and understood in order to have sex and gender based medicine more widely adopted into health profession education. Recognizing and understanding these concepts provides a foundation for developing practical approaches to incorporate SGBM information throughout existing curricula. The SGME Summit was planned with the goals of increasing participants’ awareness of the current level of knowledge regarding sex and gender differences, identifying areas where additional research is needed, highlighting gaps in medical education, providing educational resources to assist with the integration of sex and gender evidence into medical school curricula, promoting sex and gender networks, and advocating for this change. Discussions about existing curricula and teaching materials, in particular, provided practical examples of how and where this material could be included in both didactic and clinical activities. Results showed that participants perceived the Summit as valuable, both in increasing their understanding of SGBM and in providing them with resources to integrate SGBM into medical education at their respective institutions.

Critical to implementing curricular change is recognizing potential obstacles that would slow the process. LCME accreditation standards may be perceived as an obstacle. However, incorporating SGBM content into curricula can actually fulfill LCME requirements which may facilitate its adoption by medical schools. Other obstacles identified during the Summit included how to engage faculty and medical school and curricular leadership. The ultimate goal of the Summit is to encourage and facilitate adoption of dedicated SGBM education curricula into all medical schools within the next 5 years.

## Conclusion

The 2015 SGME Summit represents a first of its kind event, focused on sex and gender evidence integration in medical school education. Building upon a foundational premise of quality curricular development, the Summit program included national leaders in medical education working side by side with academic clinicians, educators, and researchers, bringing an evidence-based approach to SGME. The pre- and post-surveys confirmed that attendees were positively impacted and their knowledge, attitudes, and awareness altered by this educational experience. It would be shortsighted to believe that this educational event was enough to ensure that sex and gender evidence will be integrated throughout US medical schools. Much work remains, but the models presented during the Summit, including those that thread sex and gender into existing curricula, as well as providing model educational resources, will help advance this initiative. In addition, we will continue to work with accreditation and health professional licensure entities, student and faculty professional organizations, SGME Summit attendees, deans, and sponsors. Future efforts will also include engaging with interprofessional education efforts to launch SGBM across academic health sciences centers.

## References

[1] Council on Graduate Medical Education. Fifth report: women & medicine: physician education in women’s health and women in the physician workforce. Washington, DC: U.S. Department of Health and Human Services, Public Health Service, Health Resources and Services; 1995 July. Pub. No. HRSA-P-DM-91-1.

[2] Wizemann TM, Pardue ML (2001). Exploring the biological contributions to human health: does sex matter? Board on Health Sciences Policy.

[3] NIH Guide. NIH Guidelines on the inclusion of women and minorities as subjects in clinical research. 1994;23(11). Available from: http://grants.nih.gov/grants/guide/notice-files/not94-100.html [Accessed 16 May 2016].

[4] Our introductory discussion of sex and gender refers to both variables as a binary system. However, intersex is an another category for sex in addition to male and female. Kaneshiro NK. Intersex. Bethesda: U.S. National Library of Medicine MedlinePlus Medical Encyclopedia. [cited 2016 Jan 12]. Available from https://www.nlm.nih.gov/medlineplus/ency/article/001669.htm.

[5] Connell RW (2002). Gender. Cambridge U.K and Malden.

[6] Gender identity falls along a continuum and includes transgender, while gender expression varies based on social context. Bockting WO. From construction to context: gender through the eyes of the transgendered. SIECUS Report. 1999;1:3–7.

[7] McSweeney JC, Cody M, O’Sullivan P, Elberson K, Moser DK, Garvin BJ (2003). Women’s early warning symptoms of acute myocardial infarction. Circulation.

[8] Khan NA, Daskalopoulou SS, Karp I (2013). Sex differences in acute coronary syndrome symptom presentation in young patients. JAMA Intern Med.

[9] Mehta LS, Beckie TM, DeVon HA, Grines CL, Krumholz HM, Johnson MN, Lindley KJ, Vaccarino V, Wang TY, Watson KE, Wenger NK, American Heart Association Cardiovascular Disease in Women and Special Populations Committee of the Council on Clinical Cardiology, Council on Epidemiology and Prevention, Council on Cardiovascular and Stroke Nursing, and Council on Quality of Care and Outcomes Research (2016). Acute myocardial infarction in women: a scientific statement from the American Heart Association. Circulation.

[10] Haughom BD, Erickson BJ, Hellman MD, Jacobs JJ (2015). Do complication rates differ by gender after metal-on-metal hip resurfacing arthroplasty? A systematic review. Clin Orthop Relat Res.

[11] Maas AH, Appelman Y (2010). Gender differences in coronary heart disease. Neth Heart J.

[12] von Friesendorff M, McGuigan FE, Besjakov J, Akesson K (2011). Hip fracture in men—survival and subsequent fractures: a cohort study with 22-year follow-up. J Am Geriatr Soc.

[13] Government Accountability Office. Drug safety: most drugs withdrawn in recent years had greater health risks for women. Washington, DC: U.S. Department of Health & Human Services; 2001 Jan. Report No.: GAO-010286R.

[14] Data from Marjorie Jenkins, MD, Texas Tech University Health Sciences Center. Manuscript under preparation.

[15] Miller VM, Rice MR, Schiebinger L, Jenkins MR, Werbinski J, Nunez A, Wood S, Viggiano TR, Shuster LT (2013). Embedding concepts of sex and gender health differences into medical curricula. J Womens Health.

[16] Jenkins M, Chin EL, Rojek M, Edgell D, Hoggatt M, editors. Sex and gender medical education summit: a roadmap for curricular innovation. Program Proceedings; 2015 10.1186/s13293-016-0091-9 PMC5073999 27790364

[17] McGregor AJ, Núñez A, Barron R, Casanova R, Chin EL. Utilization of sex and gender based medical education resources and creating student competencies: a summit workshop summary. Biology of Sex Differences. doi:10.1186/s13293-016-0092-8. 10.1186/s13293-016-0092-8 PMC5073901 27785345

[18] Ludwig S, Oerfelt-Prigione S, Kurmeyer C, Gross M, Gruters-Kieslich A, Regitz-Zagrosek V, Peters H (2015). A successful strategy to integrate sex and gender medicine into a newly developed medical curriculum. J Womens Health.

[19] Kotter JP (2013). Leading change, with a new preface by the author.

